# A Multitask Learning Approach for Intrusion Detection in Controller Area Networks

**DOI:** 10.3390/s26113432

**Published:** 2026-05-29

**Authors:** Bianca Brişan, Camil Jichici, Raul Robu, Bogdan Groza

**Affiliations:** Faculty of Automatics and Computers, Politehnica University of Timisoara, 300223 Timişoara, Timiş, Romania; bianca.buleu@student.upt.ro (B.B.); raul.robu@upt.ro (R.R.); bogdan.groza@upt.ro (B.G.)

**Keywords:** intrusion detection, CAN bus, multitask learning

## Abstract

Intrusion detection on in-vehicle networks requires high accuracy, which is reported by many papers so far, but also computational efficiency to make it suitable for real-world scenarios. The achievement of both requirements at the same time becomes harder to achieve, especially as the number of attacks diversifies. An approach to leverage computational costs is the use of sliding windows, i.e., batch processing, which extends the detection over multiple frames, but the use of multitask learning is also advantageous because a number of layers are shared between classes to extract common relevant features. While indeed the greatest computational gains are from the use of a sliding window, multitask learning has benefits too and is in fact necessary as multiple attack types can coexist in the same window. We explore the benefits of this approach on three existing attack datasets and we also build our own dataset that garners more attack complexity so that we can concretely measure the benefits of multitask learning both in terms of detection rate and computational savings. Our approach considers the feature-level similarity between attack types and legitimate frames, extracted from the mutual information between the two, and extends detection over windows of multiple frames, which justify multitask learning as frames belonging to different classes can co-exist in the same window.

## 1. Introduction

In-vehicle networks have been subject to a significant number of attacks in recent years and there is an over increasing demand for designing in-vehicle intrusion detection systems (IDS). The integration of intrusion detection systems faces numerous drawbacks, such as the resource limitations inside vehicles, low-processing power and limited storage, as well as the criticality of real-time responses—latencies are critical and generally in the order of milliseconds or less. However, the complexity of the attacks, the fact that many of them are not known, e.g., zero-days, or result from the combination of more than one attack, is not the last of the concerns. In this context the use of deep neural networks has been highly pressed recently, although it comes at exquisite costs sometimes.

To alleviate some of the drawbacks regarding the costs of machine learning, multitask learning (MTL) can contribute to some extent, in contrast to single-task learning (STL). On the one hand, it saves some computational costs by relying on shared layers for feature extraction, reducing both the memory footprint and the computational load. On the other hand, MTL can also improve performance in dealing with unknown attacks by learning the behavior of the normal class and refining the deviations via auxiliary tasks. It also mitigates data imbalance, which is common in intrusion detection where legitimate packets are far more abundant, by reducing overfitting on minority attack classes through the regularizing effect of auxiliary tasks.

In this work, we build on MTL as the core concept behind our IDS methodology. Our design choices are made judiciously, considering feature-level mutual information, leveraged via the cosine similarity and the Jaccard index, as support for the similitude between the various types of attacks and legitimate frames.

We extend the detection over windows of multiple frames, which was in fact done by many previous works, justifying the need for multitask learning from the fact that frames from multiple classes can now co-exist in the same window. Note that this is not the case when doing the detection over single frames since one frame will either be legitimate or belong to a single attack class. The similitude between window labels is also assessed using the cosine similarity and the Jaccard index. As a result of our approach, the detection speed improves due to both multitask learning and the window size, and the improvement is relevant because of the mere real-time nature of in-vehicle traffic.

**Figure 1:**
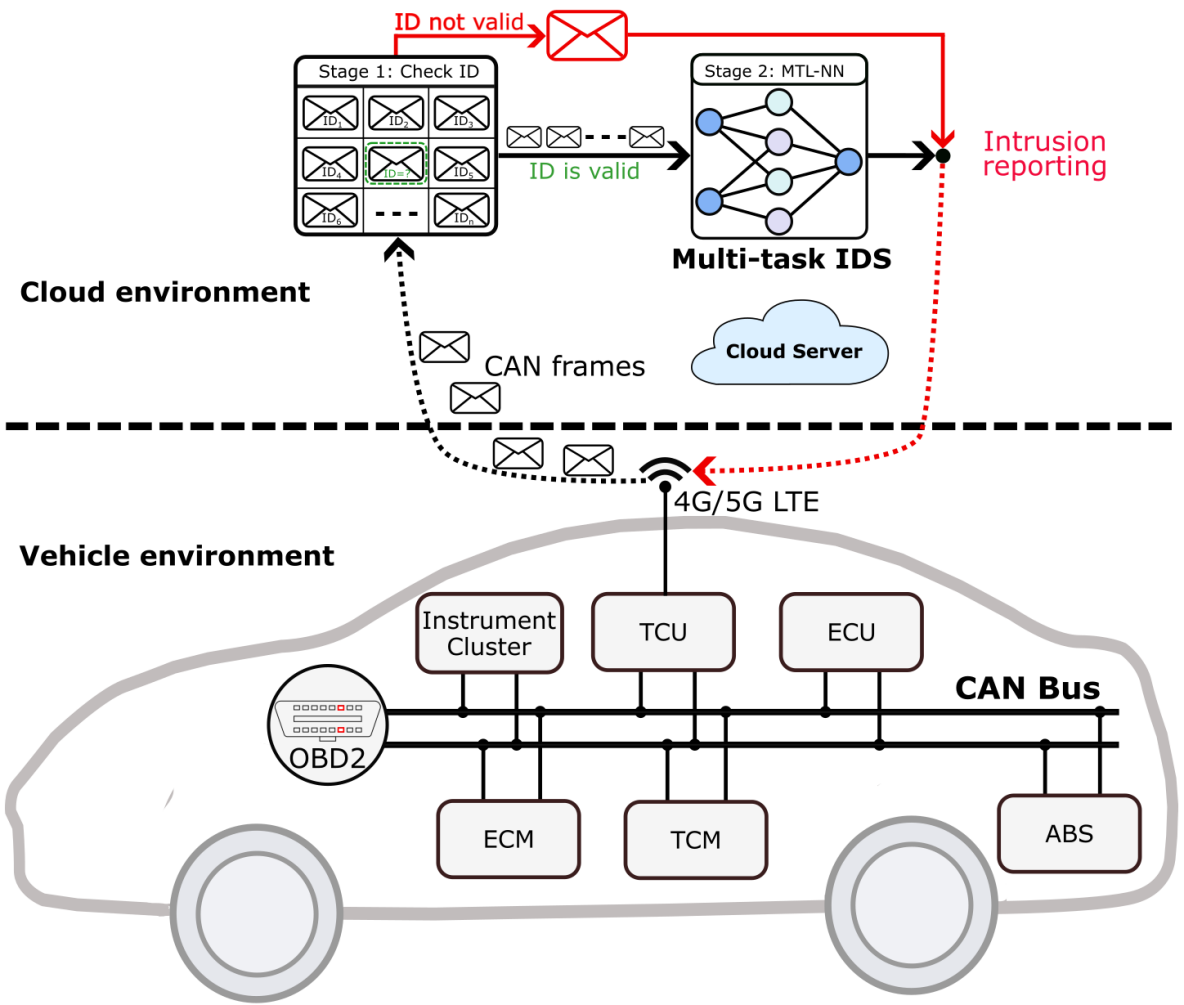
Suggestive depiction of our IDS proposal.

Figure 1 shows an overview of the system that we envision. We are generally targeting a cloud-based IDS that collects data from a vehicle and uses a multitask IDS for attack classification. Deploying the proposed MTL-based IDS in the cloud environment is advantageous because it demands computational resources beyond typical ECU capabilities [1], enables transfer learning so that knowledge of attacks detected in one vehicle can improve detection in others [2], and supports centralized updates and collection of data from multiple vehicles to enhance the detection of multiple attacks while providing secure and standardized management of detection reports in line with industry standards [3]. The choice to deploy in the cloud is also motivated by industry considerations. According to Vector Informatik, a leading global provider of software and hardware components for automotive networks, modern intrusion detection systems in vehicles are built as distributed solutions combining ECU-level monitoring with cloud-based analysis. This setup centralizes security data, enhances detection through shared insights, and simplifies updates, making security management more efficient [4]. Alternatives for local deployment may use high-end in-vehicle controllers or modern Android head-units [5], but the cloud-based IDS is centralized and can operate with data collected from multiple vehicles.

The contributions of this work can be summarized as follows:

- We propose a multitask learning approach for detecting attacks on the CAN bus grounded in an analysis of inter-class relationships using feature-class mutual information, quantified via cosine similarity and the Jaccard index.
- We construct a dataset, to be made publicly available for future research, that gathers the most significant types of attack from three existing datasets and implements them on a CAN trace from a vehicle with the help of CANoe version 11.0 SP2, an industry standard tool.
- We demonstrate that the detection time improves by a factor of around 2 times due to the use of MTL, compared with STL, and up to 6.5–9.1 times by increasing window sizes from 1 to 8 CAN frames, which is preserved for STL too, without significant degradation of detection performance.

The rest of the paper is organized as follows. In Section 2, we present the related works on intrusion detection and multitask learning. Section 3 presents some background on CAN and the adversary model. Section 4 describes the datasets and pre-processing steps. In Section 5, we present the approach from our work, while in Section 6 we show the experimental results. Finally, Section 7 holds the conclusion of our work.

## 2. Related Work

The increased connectivity in modern vehicles has exposed in-vehicle networks to serious security risks, as various studies have identified attack surfaces and demonstrated a wide range of attacks targeting safety-critical vehicle components [6,7,8]. Recently, attacks have become more sophisticated and researchers have demonstrated that they can be mounted remotely. For example, the authors of [9] manipulated ECUs such as the gateway, body control module (BCM), and autopilot by exploiting the over-the-air (OTA) software update mechanism of Tesla cars. A more recent study proposes the CANdid attack [10] which exploits the UDS services over CAN and shows how a compromised non-critical ECU can reprogram critical ones. In [11], the authors demonstrate that Electronic Logging Devices (ELDs) employed in commercial trucks can be wirelessly exploited to send arbitrary CAN messages, enabling unauthorized control over vehicle systems. Another work [12], demonstrates that smart glasses can be exploited to remotely control critical functions in Tesla vehicles, highlighting the need for security countermeasures. A detailed overview of the security vulnerabilities of the CAN protocol and its attack surface for real-world implementations can be found in [13,14].

To mitigate these attacks, both researchers and the industry have proposed numerous security solutions. Initially, the research community concentrated on using cryptographic message authentication codes (MACs) [15,16] to secure the CAN bus. Similarly, after a few years, in 2020, the Automotive Open System Architecture (AUTOSAR) released the Secure On-Board Communication (SecOC) standard [17], which also relies on MACs. A more recent trend is the design and development of intrusion detection systems (IDSs) to monitor and protect the CAN bus. Due to its high relevance, numerous IDS solutions employing various detection approaches were proposed. Recent surveys offer an comprehensive overview on attacks and intrusion detection systems [18,19,20]. The use of Bloom filters to detect anomalous CAN bus traffic was explored in [21], other works account for the use of entropy [22,23], the offset ratio for responses triggered by remote frames [24], or Hamming distances [25,26] to detect CAN bus attacks.

Conversely, many IDS solutions rely on machine learning algorithms, as surveyed in [27,28]. Recent literature indicates a trend towards deep learning models, capable of automatically capturing spatiotemporal relationships between CAN messages, without requiring manual definition of rules or features [29,30]. These models allow the system to learn normal network behavior directly from data and identify subtle deviations that may signal abnormal activity, without depending on predefined thresholds or manually constructed heuristics. Various works have focused on improving the feasibility of implementing these solutions in real-world scenarios. Some approaches propose lightweight models or parallel architectures to reduce computational cost and allow the integration of the IDS directly at the ECUs level [31].

Other directions explore federated learning, which allows collaborative training of models without sharing raw data. Federated learning allows training of a common intrusion detection model directly in vehicles, keeping CAN traffic data localized at vehicle level, and transmitting only the model parameters to a server for centralized aggregation [32,33]. Attention mechanisms were also introduced to improve the model’s ability to capture subtle relationships between CAN messages and highlight relevant sequences in temporal context, leading to more sensitive and robust anomaly detection [29,34]. In [35], the semantic knowledge of a BERT model [36] is distilled into a lighter CNN–BiLSTM architecture, reducing computational cost without substantially compromising detection performance. The authors from [37] propose LETNN (Locally Enhanced Toeplitz Neural Network), a model that combines the efficiency of TNN (Toeplitz Neural Network) with a local analysis mechanism called LBCO (Local Binary Comparison Operator), which compares message positions to identify very subtle attacks. Experimental results have demonstrated computational efficiency, high accuracy, and low false alarm rate (FPR). Cosine similarity has also been explored in prior works [38,39] for CAN bus intrusion detection. In [38,39], the authors use cosine similarity for detection purposes, while in our work we do not use it for detection but only to justify the similarity of the feature representations for multitask learning.

However, only a small number of works explore the application of multitask learning (MTL) techniques to improve the performance of IDSs. The main approaches regarding the detection of attacks on the CAN network using MTL are presented in what follows. In [40], the authors compare multitask and multi-stage learning approaches for CAN message classification using ANN, LSTM and CNN models. While both strategies achieve similar accuracy, the multi-stage approach yields better precision and recall in multi-class attack classification, with the best performance obtained by a CNN model (98% accuracy for binary and 99% for multi-class classification). Another work [41] addresses the problem of anomaly detection in imbalanced scenarios, where certain rare maneuvers (such as U-turns) may be erroneously classified as anomalies by traditional models. A multitask learning framework is proposed in which an autoencoder for anomaly detection and a maneuver prediction network share a common convolutional Bi-LSTM encoder. By jointly learning reconstruction and driving-behavior prediction, the model reduces false alarms. The authors of [42] propose an IDS for CAN bus based on deep learning and multitask learning. Their model (CANLite) captures both spatial and temporal relationships between sensors and was trained and tested on two datasets, achieving an attack detection rate of over 95%. The results obtained were compared with those obtained by the NeuroCAN reference model [43]. The multitask learning model obtained similar results to the reference model but with a memory footprint 50% smaller than the reference model. In [44], the authors propose a multitask learning model that has two outputs: one output classifies the type of attack (benign, DoS, fuzzing and impersonation) and the other output classifies the ECUs identifying the attacker. The proposed model uses the unique physical signatures of the voltage signals generated by each ECU. Using hardware imperfections and signal variations, the model can differentiate ECUs, even if the attackers use valid IDs. The multitask approach ensures a reduction in detection latency. Another work [45] presents a real-time IDS that employs a multitask model based on GRU (Gated Recurrent Units), along with the dynamic label watermark (DLW) technique applied to CAN frames to protect the system against conventional and adversarial blackbox attacks. The model is fast, compact, accurate and outperforms or matches the performance of reference models (LSTM, GRU, CANLite, ConvLSTM-GNB) across all tested scenarios. In [46], the authors propose a literal multi-dimensional anomaly detection method for Controller Area Networks (CANs), employing the distributed long-short-term-memory (LSTM) framework. The method uses multitask LSTM neural networks deployed on mobile edges to detect anomalies from both the time and data dimensions at the same time, improving detection efficiency and accuracy.

## 3. Background on CAN and Adversary Model

This section presents the CAN protocol and the adversary model, also giving details on how our data set was built.

### 3.1. CAN Basics

From its introduction by Robert Bosch in the 80 s [47], the Controller Area Network (CAN) bus has demonstrated its robustness, reliability, and cost efficiency. Because of these strengths, the CAN bus is still the preferred solution by original equipment manufacturers (OEMs), to allow communication between Electronic Control Units (ECUs) throughout a vehicle. The high-speed CAN protocol permits the transfer of 8 bytes per frame at a bit rate of up to 1 Mbit/s.

**Figure 2:**
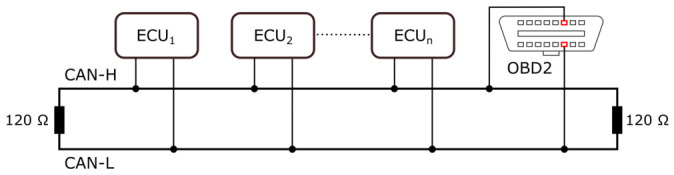
Typical CAN network architecture.

A typical CAN network architecture is suggested in Figure 2. The CAN bus has a straightforward electrical design, consisting of two wires, CAN-High (CAN-H) and CAN-Low (CAN-L), both ending with a 120 Ω resistor. As shown in Figure 2, each node or interface (e.g., a diagnostic interface like OBD2) connects to the CAN bus through a stub that links the two wires: CAN-H and CAN-L. The exchange of actual data (vehicle parameters) between the ECUs is facilitated by data frames. Since CAN is a broadcast communication protocol, every node in the network can receive every transmitted data frame, but more often than not an ID filtering mechanism is employed and only those ECUs interested in the specific ID will use the specific frame.

**Figure 3:**
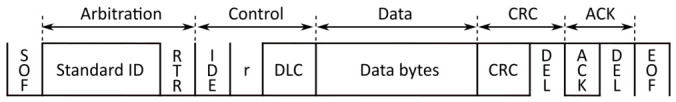
Standard CAN data frame.

Figure 3 illustrates the field organization inside a CAN data frame. A CAN frame transmission is indicated by a Start of Frame (SOF) bit encoded as a logical 0 (dominant state). Next, the message Identifier (ID) and the Remote Transmission Request bit (RTR) are included in the arbitration field. The RTR bit exhibits a dominant state for data frames or a recessive state for remote frames (which are requests for the actual data). Each CAN frame carries an 11-bit ID in case of the standard format, while for the extended format the ID has a length of 29 bits. Apart from its function that uniquely identifies a CAN frame, the CAN ID also guarantees the bus arbitration process, meaning that if several nodes attempt to occupy the bus simultaneously, the node that sends the ID with the lowest value wins the arbitration. The control field includes three parts: an Identifier Extension bit (IDE), a Reserved bit (r), and the Data Length Code (DLC). When using 11 bit IDs (standard format), the IDE bit is encoded as a logical 0 (dominant state), whereas for 29 bit IDs (extended format), it is encoded as a logical 1 (recessive state). The most important relevant part of the control field, the DLC, indicates how many data bytes are transmitted by the actual frame inside the data field. To ensure data integrity and protect communication against unintended modifications, such as those caused by electrical noise, a 15-bit Cyclic Redundancy Check (CRC) code is calculated based on the message content. This error detection code verifies that the transmitted data remain uncorrupted. Upon successful reception of a CAN frame, each node acknowledges it by transmitting a dominant bit (logical 0) in the Acknowledge (ACK) slot while if no such acknowledgment is sent, the slot remains in its default recessive state (logical 1). The message transmission ends with a predetermined sequence of 7 consecutive recessive bits grouped as the End of Frame (EOF) field.

### 3.2. Adversary Model

Existing works on CAN IDS, usually account for the following attack types: malfunction, fuzzing, spoofing, replay, and flooding (DoS). To comply with this, we add these attacks on data collected from a recent paper [2] and, thus, our adversary model includes the following:

- *Malfunction attacks*: the adversary monitors the CAN bus, captures a genuine frame, and injects a crafted message with the same ID and a randomly generated payload.
- *Fuzzing attacks*: this attack type is similar to the previous one, the only difference is that the CAN ID is randomly generated between 0 (0x000) and 2047 (0x7FF); however, if the randomly generated ID happens to be part of the legitimate IDs, the attack is not mounted to avoid reproducing the same behavior as in the malfunction attack.
- *Spoofing (vehicle speed)*: the adversary targets frames carrying the vehicle speed signal, captures one of these frames, and injects a crafted message with the altered speed data (for our trace, we empirically determined that vehicle speed is carried by two CAN frames, each containing speed signals for two individual wheels).
- *Replay attacks*: the adversary listens to the CAN bus, records a legitimate frame, and injects the exact same message on the bus at a later time.
- *Flooding*: the adversary sends bursts of 10 attack frames carrying ID 0x000 with 150 μs between them to flood the CAN bus.

For a clearer comparison, Table 1 summarizes these adversarial behaviors and how they are addressed in various datasets from earlier research on CAN IDSs [2,48,49], as well as in our dataset.

The steps involved in the generation of our own data are described next. The attack trace was built using CANoe, a widely recognized tool used extensively in the automotive industry to simulate vehicle network communications. Our simulation designed in CANoe environment for generating the attack trace consists of two nodes. The first is a replay block, a special node that reproduces the recorded CAN traffic from the target vehicle. The second is a CAPL (Communication Access Programming Language)-based node that emulates the behavior of an adversary by intercepting the replayed CAN traffic and injecting crafted attack frames. CAPL is a programming language similar to C, specifically designed to be used in the CANoe environment. Apart from the standard C syntax and logic, it provides extended functionalities tailored to CANoe, such as support for event-driven programming, system variables, definition or manipulation of message structures, and integration with communication databases (e.g., DBC files). These features allow for flexible network traffic monitoring, simulation, and control.

Additionally, we build our dataset guided by two main objectives. The first is to include every attack type identified in related works [2,48,49] in a single trace. This approach enables us to highlight the multitask learning and detection capabilities of the neural network when it is exposed to a variety of attack patterns at the same time. The second objective concerns adversary behavior. Different from the approaches in [48,49], where the attacks are deterministic (at fixed periods), or in [50], which relies on synthetic CAN data (both legitimate and attack frames are artificially generated), our attack strategy focuses on modeling the adversary to operate probabilistically, introducing variability and unpredictability rather than mounting attacks at predetermined cycle times. This choice is motivated by the fact that deterministic attacks produce easily detectable patterns in CAN traffic due to its periodic nature. In contrast, probabilistic attack models better reflect more realistic adversarial behavior, where attacks may occur irregularly over time. Thus, the exact location of the attack in the trace is probabilistic and cannot be predicted. The legitimate CAN traffic used for the generation of our attack traces was collected from a real-world vehicle operating under practical driving conditions, including acceleration, braking, and lighting actions performed during data collection. The attack frames were subsequently injected in the CANoe environment, an industry-standard automotive network simulation tool. The CANoe platform accurately reproduces CAN communication behavior in the presence of attacks, taking into account message priorities, delays, order of CAN frames, transmission timing, and the bit-stuffing mechanism. This approach enables the generation of attack traces without directly injecting attack frames into the target vehicle, thereby avoiding potential damage while still preserving real-world CAN network traffic behavior.

The details of the attack probabilities are presented below. For each genuine CAN frame, we assign an attack probability Pr(Attack). In our simulation environment, we chose Pr(Attack)=0.25. We denote and generate the attack type as a random integer variable λ∈{0,1,2,3,4}, where each value corresponds to an adversarial behavior as outlined below:

$$\lambda =\left\{\begin{array}{c} 0,\mathrm{malfunction}\\ 1,\mathrm{fuzzing}\\ 2,\mathrm{spoofing}\;-\;\mathrm{vehicle}\;\mathrm{speed}\\ 3,\mathrm{replay}\\ 4,\mathrm{flooding} \end{array}\right.$$

Based on the generated value λ, an attack frame will be sent at a time randomly chosen between 0 and the message cycle after receiving the genuine frame for the first three types of attack. For replay attacks we leave a longer time frame of between 0 and 10×CycleTime since replays will likely have no effect over short time periods. Also, for flooding attacks we transmit a burst of 10 attack frames.

## 4. Dataset Description and Preparation Workflow

In this section, we present the four datasets that we use in this work, three of them coming from existing papers and the fourth one being designed by us specifically for this paper. Since each dataset has its own format, specific pre-processing steps were required before the data is fed into the neural network. These steps are described in what follows.

### 4.1. Car Hacking Dataset

This dataset [48] contains a CSV file for each attack type: fuzzing, spoofing (gear or RPM) and DoS. The dataset was built by logging the CAN traffic through the OBD-II port of a real-world vehicle while performing these attacks. Each CSV file records roughly 30–40 min of CAN traffic and approximately 300 attack instances, with an instance lasting between 3 and 5 s. The data files have the following format: Timestamp (time in seconds), CAN ID in hexadecimal, DLC (data length which is between 0 and 8), Data [0–7] (payload bytes) and a Flag indicating whether a CAN frame is an attack (T) or genuine (R).

**Figure 4:**
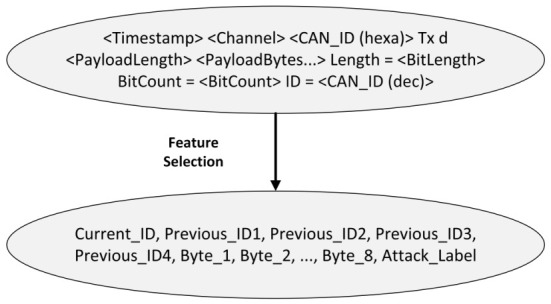
The conversion script applied to our dataset.

For each CSV file, the data pre-processing followed a consistent procedure to ensure uniformity across all data files. Initially, a small sample of rows was read without headers to identify the exact number of columns and understand the format of CAN frames from this dataset. Based on the inspection, we defined meaningful column names focusing on essential features such as the CAN IDs, data bytes and flag to distinguish between genuine or attack frame. Subsequently, each CSV file is transformed using a script into another CSV file with the following format: Current_ID, Previous_ID1, Previous_ID2, Previous_ID3, Previous_ID4, Byte_1 to Byte_8, Attack_Label, as shown at the bottom of Figure 4. We extract the current ID along with the history of the last four IDs, so that the model can learn sequential patterns of CAN IDs in the traffic, and any deviation from these sequences can be flagged as anomalous. This pre-processing step was performed according to the following rules. If the DLC of a frame is less than 8, the data field is padded with zero values up to 8 bytes, ensuring that all samples have the same input size. Since the CAN ID and payload bytes are represented in hexadecimal format, these values are converted into decimal integers. The flag column was initially encoded to distinguish between normal and attack messages (T or R). Subsequently, to identify the type of attack present in each dataset, we applied a specific labeling scheme. Attack types were mapped as follows: 0—no attack, 1—fuzzing, 2—spoofing (gear or RPM), 3—DoS. Each data file was updated with the corresponding attack label based on its category.

Finally, all data files were concatenated sequentially to create a unified data file for the model. This step, which combines all attacks into a single file, is required to highlight the multitasking capability of our neural network. The final CSV file, containing both normal and attack frames with clearly defined labels, is split so that 80% of the attack samples are in the training set and 20% are in the testing set. Afterwards, we use this file as input to train the machine learning model and to evaluate the performance of the proposed solution in detecting attacks.

### 4.2. Survival Analysis Dataset

The CAN traffic of this dataset [49] was collected from three different vehicles: Hyundai YF Sonata, KIA Soul and Chevrolet Spark, resulting in three separate datasets, one for each vehicle. Because the detection results obtained for the Chevrolet Spark vehicle were poorer than those from the Hyundai YF Sonata and KIA Soul vehicles [49], we use only the dataset logged from the Spark vehicle in our evaluation. As in the previous dataset, the attacks were also mounted by injecting malicious CAN packets during vehicle operation. The following adversarial manipulations are addressed in this dataset: malfunction, fuzzing and flooding. Compared to the previous dataset, where each CSV file corresponding to a specific attack has 30–40 min of CAN traffic, this dataset has substantially less CAN traffic, with under 1 min for each attack scenario. Besides these attack scenarios, each stored in a TXT file, this dataset also contains one TXT file recorded during normal driving. This file was not used in our evaluation, as it does not contain any attack frames. The format of each data file shares a similar structure as the Car Hacking Dataset: Timestamp (time in seconds), CAN ID in hexadecimal, DLC (data length which is between 0 and 8), Data [0–7] (payload bytes) and a Flag indicating attack (T) or genuine (R) messages.

As with the Car Hacking Dataset, we apply identical pre-processing steps: padding zero values in the payload up to 8 bytes, converting hexadecimal values to decimal and assigning specific attack labels for each adversarial manipulation and 0 for genuine frames. In the final step, all TXT files containing attacks were merged, split into training and testing sets as in the previous dataset, and fed to the neural network.

### 4.3. Cloud IDS Dataset

This dataset focuses exclusively on two attack types: fuzzing and replay. The CAN traffic from this dataset [2] was collected from three distinct vehicles of the same car model (Dacia Duster). Depending on the attack type, the dataset with attacks is structured into three folders: Fuzzing (containing data files with fuzzing attacks), Replay (containing data files with replay attacks), and Combined (containing data files with both fuzzing and replay attacks). Within each folder, there are three subfolders corresponding to Vehicle A, Vehicle B, and Vehicle C. In our experiments, we only use a data file with combined fuzzing and replay attacks from Vehicle A. We choose the one that contains more attacks targeting CAN frames with different IDs.

The structure of the dataset differs significantly from previous ones but is similar with the format employed in this work. The dataset files include the current CAN Frame ID, the IDs of the previous four CAN frames, the data field of the current CAN frame and a label indicating whether the frame is legitimate (0) or malicious (1)—the attack type is specified in the file name. The only preprocessing step remaining is to apply our specific labeling attack scheme, marking fuzzing attacks as 1 and replay attacks as 2. Therefore, based on the file name, we can identify the type of attack associated with specific CAN IDs. For example, in the file named Vehicle A—Replay 0x181, 0x161, 0x1a5, Fuzzing 0x244, 0x284, 0x354—IDs_Datafield_Classification.csv, frames with CAN IDs 0x244, 0x284, and 0x354 marked with 1 correspond to fuzzing attacks, while those with CAN IDs 0x181, 0x161, and 0x1a5 marked with 1 correspond to a replay attack. Finally, the data is divided into training and testing sets, as was done in the previous datasets, and subsequently used for the model.

### 4.4. Our Dataset

Last but not least, we present the preprocessing steps employed for our dataset. Section 3.2 already mentions that our dataset contains all previous attack types and describes the steps used in their generation. This explains why this dataset is more complex than the previously presented datasets. Our dataset contains approximately 30 min of CAN traffic and is, from this perspective, comparable to the Car Hacking dataset.

After the attack trace was generated using the CANoe tool, the resulting CAN log file augmented with specific attacks follows the CANoe CAN logging format. Figure 4 presents the script responsible for converting the CANoe format into the format required for neural network processing.

At the top of the figure, we start with the original CAN log format, which includes information such as timestamp, channel, CAN ID (in hexadecimal), payload length, payload bytes, and bit count. The raw data is generally not fed directly to the machine learning algorithm, but subjected to feature engineering. Therefore, in the next step (shown in the lower part of the figure), the data is reorganized into a structured format. For each frame, we include the current CAN ID, several previous CAN IDs (to capture temporal behavior), the payload bytes (Byte_1 to Byte_8), and an attack label. We created this format for helping the model to understand better the current message and its recent context.

Each attack frame in the generated CANoe file is marked as *#attacktype_intrusion*. Based on this, the following labeling scheme was applied: 0—no attack, 1—malfunction, 2—fuzzing, 3—spoofing (vehicle speed), 4—replay and 5—flooding. Finally, the data samples are divided into training and testing sets with the same percentages as those used in previous datasets and used to train and test the model. The dataset, which includes both the CANoe format and a CSV file generated after the preprocessing steps, is publicly available to support future research and can be accessed via the following link https://www.aut.upt.ro/~bgroza/projects/mtl-ids/combined_output_dataset.zip.

## 5. Concept and Neural Network Design

This section details the rationale behind our detection methodology, including the neural network architecture employed in our IDS.

### 5.1. Methodological Justification

We justify our choice for pursuing MTL as an approach for an in-vehicle IDS, and the neural network design behind it, starting from the feature level and proceeding up to the distribution of attacks between multiple frames as follows.

*Feature-level mutual information.* Mutual information is commonly used for feature selection [51], it was also recently used in the context of multitask learning for image segmentation [52]. Here we use it only as an argument to support our design choices: the mutual information between features and labels shows a good correlation across the various types of attacks from our dataset.

For each task $T_{\alpha },\alpha \in \left\{1\ldots \tau \right\}$ corresponding to the detection of one attack type, we compute the mutual information vector for the task over all samples $I\left(T_{\alpha }\right)=[I\left(X_{1};Y_{\alpha }\right),I\left(X_{2};Y_{\alpha }\right)$, …, $I(X_{d};Y_{\alpha })]$, where $X_{i},i=1,\ldots ,d$ is the i-th feature, *d* is the total number of features, $Y_{\alpha }$ the binary label vector for task $T_{\alpha }$ and τ is the number of tasks. Note that each task acts as a binary classifier for one attack type and, thus, $T_{\alpha }$ has a corresponding binary vector $Y_{\alpha }$ defined over all samples. Then, having the mutual information vectors for tasks $T_{\alpha },T_{\beta }$ we compute their cosine similarity:

$$S_{C}\left(I\left(T_{\alpha }\right),I\left(T_{\beta }\right)\right)=\frac{\sum_{i=1}^{d}{\left[I\left(T_{\alpha }\right)\right]}_{i}\cdot {\left[I\left(T_{\beta }\right)\right]}_{i}}{\sqrt{\sum_{i=1}^{d}{\left[I\left(T_{\alpha }\right)\right]}_{i}^{2}}\cdot \sqrt{\sum_{i=1}^{d}{\left[I\left(T_{\beta }\right)\right]}_{i}^{2}}}\;$$

Here ${\left[I\left(T_{\alpha }\right)\right]}_{i}$ and ${\left[I\left(T_{\beta }\right)\right]}_{i}$ denote the mutual information associated with the i-th feature for tasks $T_{\alpha }$, $T_{\beta }$. High values for the cosine similarity means that there is common information between the features used for identifying the attacks in each task. Indeed, the cosine similarity justifies our choice by returning large values when computed over the attack pairs.

Besides this, we also use the Jaccard Index, also referred to as the Jaccard similarity coefficient, which is a metric used to determine the similarity between finite sample sets. Here we used it to determine the similarity of the features between tasks. The index is the size of the intersection of the two sets divided by the size of their union. Let $I(T_{\alpha })$ and $I(T_{\beta })$ be the two min-max scaled sets of the mutual information between sample features and labels for the two tasks $T_{\alpha },T_{\beta }$ and let $H_{\theta }\left(I\left(T_{\alpha }\right)\right)$ and $H_{\theta }\left(I\left(T_{\beta }\right)\right)$ be the two finite sets after passing them through the Heaviside function *H* with threshold θ. That is, we replace all values greater than θ with 1 and the rest with 0, binarizing the mutual information scores according to whether the feature has contributed or not above threshold τ. We define the Jaccard Index $J(H_{\theta }\left(I\left(T_{\alpha }\right)\right),H_{\theta }\left(I\left(T_{\beta }\right)\right))$ as follows:

$$\begin{array}{cc} \; & J(H_{\theta }(I\left(T_{\alpha }\right),H_{\theta }\left(I\left(T_{\beta }\right)\right)=\frac{|H_{\theta }(I\left(T_{\alpha }\right)\cap H_{\theta }\left(I\left(T_{\beta }\right)\right|}{|H_{\theta }(I\left(T_{\alpha }\right)\cup H_{\theta }\left(I\left(T_{\beta }\right)\right|}\\ \; & =\frac{|H_{\theta }(I\left(T_{\alpha }\right)\cap H_{\theta }\left(I\left(T_{\beta }\right)\right|}{|H_{\theta }(I\left(T_{\alpha }\right)\left|+\right|H_{\theta }(I\left(T_{\beta }\right)\left|-\right|H_{\theta }(I\left(T_{\alpha }\right)\cap H_{\theta }\left(I\left(T_{\beta }\right)\right|}\; \end{array}$$

The value of $J(H_{\theta }\left(I\left(T_{\alpha }\right),H_{\theta }\left(I\left(T_{\beta }\right)\right)\right)$ is always between 0 and 1 with a value of 1 indicates that the sets are identical, i.e., the tasks use identical features that contributed above threshold θ, and 0 indicates they are disjoint, i.e., no common features that contributed above threshold θ.

**Figure 5:**
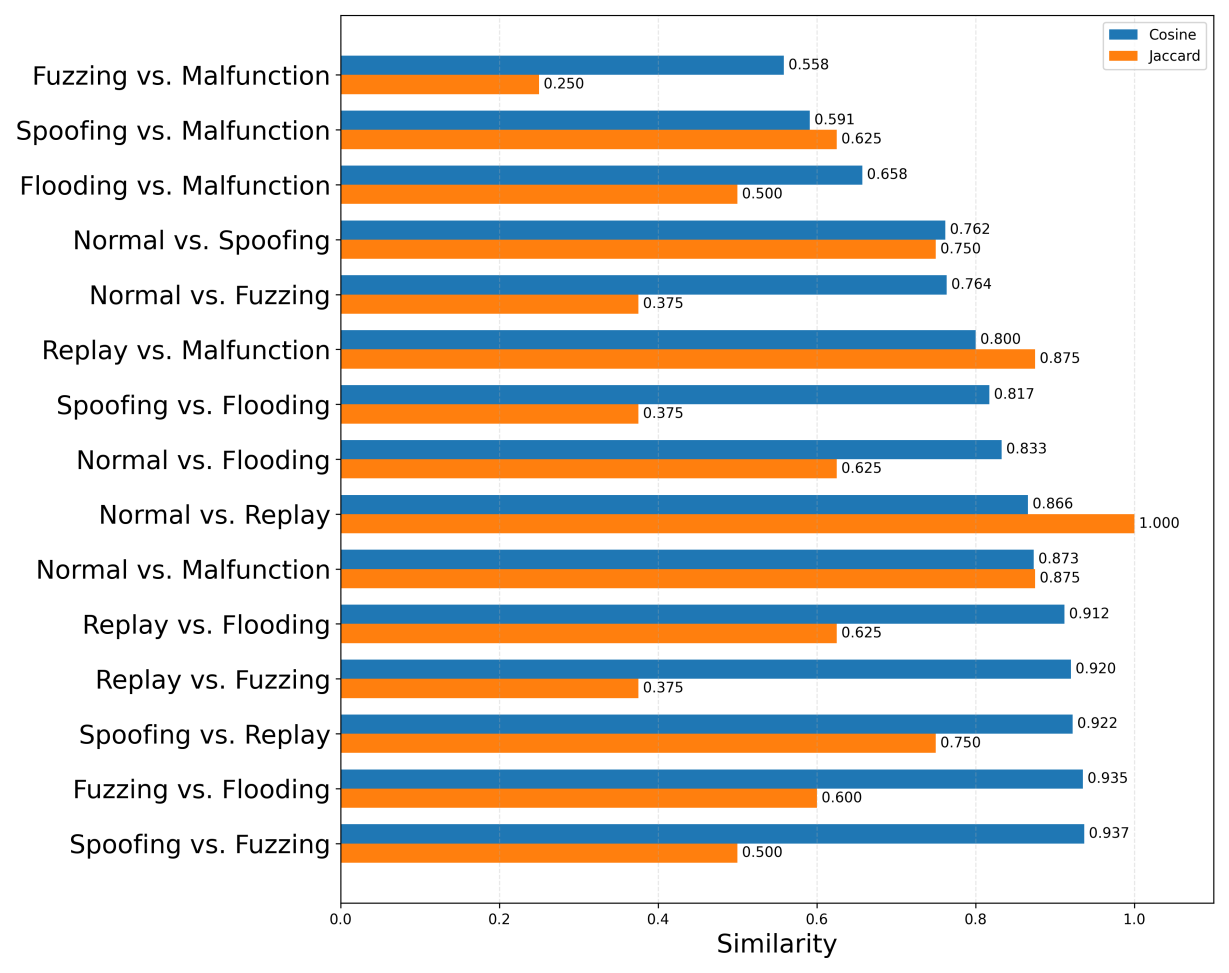
Task similarity for detecting each type of attack at feature level, computed from Mutual Information using Cosine Similarity vs. Jaccard Index.

Figure 5 shows the above similarity scores when computed over one of the attack traces that we use in this work. The scores were computed using Python 3.11.9 with scikit-learn library functions cosine_similarity and jaccard_score over the score returned by mutual_info_classif that was scaled in the [0,1] range. For this plot we set θ=0.2. The cossine similarity is high between most of the tasks, while the Jaccard index has a greater variance and the two scores complement well. For example, the normal vs. replay comparison gives a perfect Jaccard score of 1.0, which is expected since replays are an exact match for normal frames. There is a very low Jaccard score for normal vs. fuzzing, spoofing vs. flooding and replay vs. fuzzing attacks, which is due to relevant differences between these attacks, e.g., random IDs, all-zero IDs vs. regular IDs.

**Figure 6:**
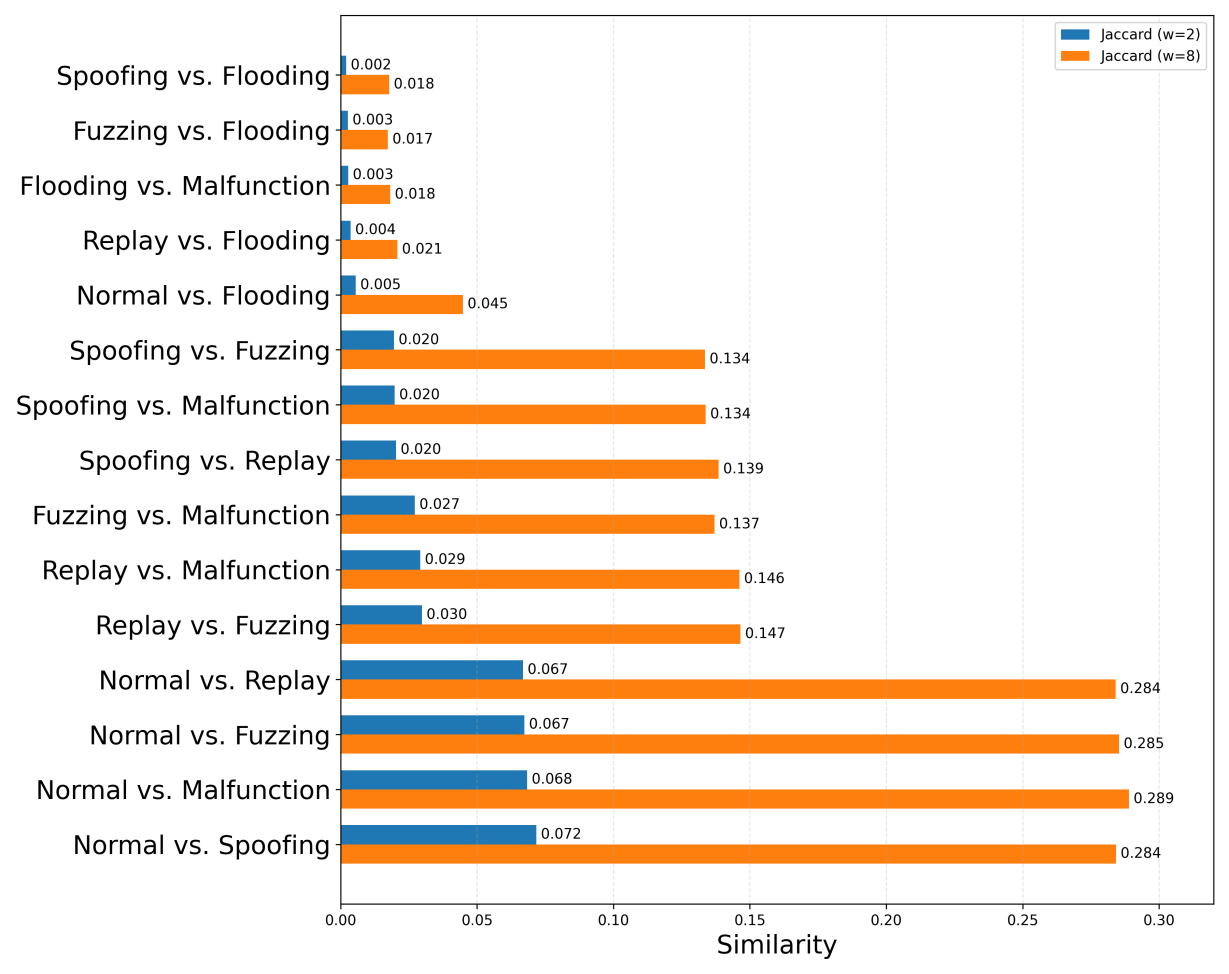
Jaccard similarity between window labels for w=2 vs. w=8.

*Window-level mutual information and label similarity.* As stated, we try to reduce the computational time by extending the detection over a window of multiple frames rather than using a single frame. When doing so, the labels on each window are mixed and the classes are no longer exclusive since each window can hold multiple attacks—this again justifies the MTL approach. However, the mutual information score computed between the resulting labels/classes was very low. This is expected since two attacks may occur at the same time, but this happens more rarely. This justifies an MTL network that has a shallow common layer and extends the heads that are used for the detection of each attack. When computing the Jaccard similarity index over the windows labels, there is an expected increase in the similarity of the labels as the window length increases. This happens because as the window size becomes larger, each window increases its chances of collecting a frame from each class (legitimate or attack). Figure 6 shows the Jaccard index contrasting between w=2 and w=8. The increase in the Jaccard index is almost linear, that is, by a factor of 4 when from w=2 to w=8. Note that the value of this index is 0 when w=1 since each window will belong to a single class.

### 5.2. Detection Methodology and Neural Neural Architecture

**Figure 7:**
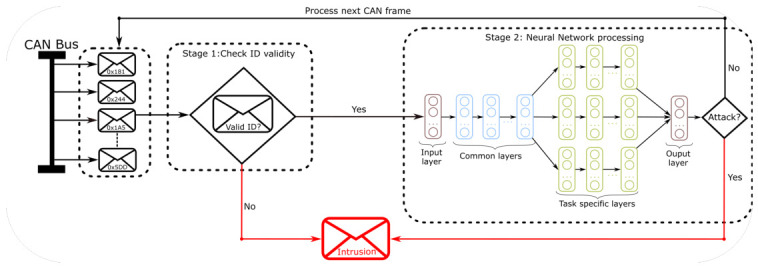
Overview of the proposed solution.

Our proposal involves a two-stage IDS as shown in Figure 7. The first stage is a filtering mechanism that processes the incoming CAN frames and checks a lookup table containing genuine CAN IDs (legitimate traffic). This is feasible given that current CAN traffic datasets rely on static IDs and it also increases efficiency since many attack datasets [48,49] use attack frames with IDs that are not part of the legitimate traffic and can be much more easily detected this way without wasting computational resources for ML algorithms. Although dynamic ID allocation has been explored in prior works [53,54], it would introduce changes to the CAN arbitration mechanism and is, therefore, unlikely to be adopted in most existing vehicles. Even in such cases, the construction of the lookup table remains straightforward. The lookup table contains CAN identifiers associated with legitimate traffic. Each incoming frame is processed by the ECU, which performs a linear search over the table to check for the presence of the corresponding ID.

Using this table, flooding attacks and a significant portion of fuzzing attacks can be easily detected, as their CAN IDs are not part of the legitimate traffic. Specifically, frames with ID = 0x000 are flagged as flooding attacks, while frames with IDs not present in genuine CAN traffic are identified as fuzzing attacks. The purpose of this stage is to reduce computational overhead by preventing adversary frames with non-legitimate IDs from being processed by the MTL stage. We also account for the number of flooding attacks and a subset of fuzzing attacks detected in this stage to enable the aggregation of performance metrics once the entire detection process, including the second stage, is finished. Frames that pass this initial filtering mechanism are forwarded to the second stage of the IDS—which employs a multitask neural network to detect malicious activity.

**Figure 8:**
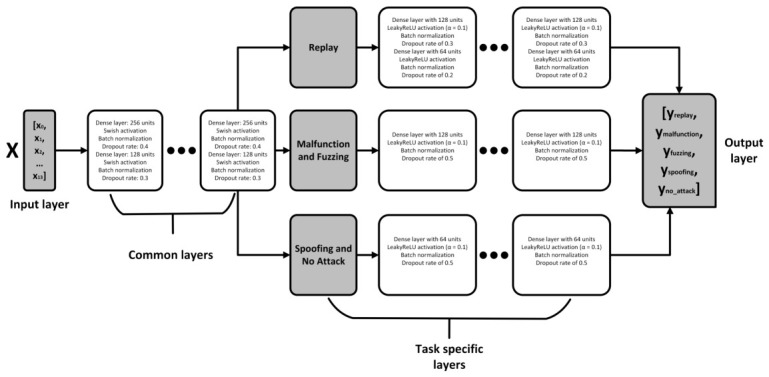
The architecture of the neural network model.

The architecture of the neural network model is detailed in Figure 8 and consists of two main parts: the shared layers (trunk) and the separate layers which are specific for each type of attack (branches). The shared layers act as a common feature extractor, collecting general features from the input data that are useful for all attack types. The branches are specialized for detecting a specific type of attack (if exists). Each branch has its own set of layers designed based on the unique characteristics of that particular abnormal behavior.

Following the architecture overview, the input layer receives a feature vector of 14 dimensions. The shared trunk consists of two fully connected layers with 256 and 128 neurons, respectively, both using the Swish activation function. Batch normalization is applied after each layer to stabilize training, while dropout rates of 0.4 and 0.3 are used to reduce overfitting. The shared representation is then forwarded to multiple task-specific branches. The replay branch is the most complex, comprising two dense layers with 128 and 64 neurons, LeakyReLU activation, batch normalization, and dropout rates of 0.3 and 0.2. The malfunction and fuzzing branches follow a similar design, each using a single dense layer with 128 neurons, LeakyReLU activation, batch normalization, and a dropout rate of 0.5. The spoofing and no-attack branches are simpler, each containing one dense layer with 64 neurons, LeakyReLU activation, batch normalization, and a dropout rate of 0.5. Each branch terminates in a single sigmoid-activated neuron, producing a probability score that indicates the presence or absence of the corresponding attack type.

## 6. Metrics and Results

In this section we define the metrics and we particularly emphasize on the recall, which is the ability of the system to recognize attacks, then proceed to the experimental results.

### 6.1. Metrics Employed for Evaluating the Detection Performance

For the proposed IDS, the model predicts exactly one label, which corresponds to a specific attack or legitimate frame. We remind the reader that in our dataset, we have the following labels: 0—no attack, 1—replay, 2—malfunction, 3—spoofed and 4—fuzzing. To evaluate how well the model distinguishes between the classes, the following metrics are used for each class individually:

- True Positive (TP): the number of times the model correctly predicts a class for a frame, where the class can be no attack or a specific attack type.
- True Negative (TN): the number of times the model correctly predicts a class different from the current one.
- False Positive (FP): the number of times the model incorrectly predicts a specific class, which can be either no attack or a specific attack type.
- False Negative (FN): the number of times the model incorrectly predicts a class different from the true one.

From the values of TP, TN, FP and FN, the following performance metrics were used for each attack class: True Negative Rate (TNR), False Positive Rate (FPR) and Recall. These are defined in the usual way as follows:

- TNR represents the proportion of frames not belonging to the considered attack class that were correctly identified as such:
  $$\mathrm{TNR}=\frac{TN}{TN+FP}\;$$
- FPR indicates the proportion of frames from other classes that were incorrectly classified as the considered attack:  
  $$\mathrm{FPR}=\frac{FP}{FP+TN}\;$$
- Recall or TPR the proportion of actual attack instances of the given class that were correctly detected:
  $$\mathrm{Recall}=\frac{TP}{TP+FN}\;$$

The ROC–AUC metric describes how well the model distinguishes between normal and malicious frames. It is based on the ROC curve, which shows the relationship between the true positive rate and the false positive rate for different decision thresholds. The area under this curve represents the model’s overall ability to correctly separate positive samples from negative ones. A higher ROC–AUC value indicates that the model is more effective at distinguishing attacks from normal behavior, while a value close to 0.5 suggests performance similar to random guessing.

The PRC–AUC metric evaluates the balance between precision and recall and focuses on how well the model identifies positive samples. It is derived from the precision–recall curve, which illustrates the trade-off between correctly detected attacks and false alarms across different thresholds. The area under this curve summarizes the model’s effectiveness in detecting attacks while maintaining reliable predictions. PRC–AUC is particularly important in imbalanced datasets, where attack samples are much fewer than normal ones, as it better reflects the model’s true detection capability.

In light of the aforementioned datasets and the above metrics, we proceed to presenting the experimental results.

### 6.2. Experimental Results

For our experiments, we used an Apple Macbook Pro (Apple, Cupertino, CA, USA) equipped with a 12-core M3 Pro processor and 18 GB of RAM, running macOS 26.2. The proposed neural network was evaluated on the four different datasets using the same experimental protocol. The input data were grouped into fixed-size temporal windows composed of consecutive CAN frames, with window sizes ranging from one frame per window up to 32 frames. This setup allows the analysis of how different amounts of temporal context influence attack detection performance.

Table 2 summarizes the main hyperparameters from our experiments for training the proposed model, including the optimizer configuration, learning rate, batch size, number of epochs, and regularization techniques.

Based on the results obtained across all datasets, it was observed that a window size of 8 frames provides the best overall trade-off between detection accuracy and computational efficiency. While smaller windows lead to reduce performance gains, larger windows (16 and 32 frames) lead to a decrease in detection performance, particularly for certain attack types. This trade-off is normal and expected. Therefore, the most relevant window sizes, i.e., 1, 2, and 8 frames, are presented in detail in this section. Extended results for larger window sizes, i.e., 16 and 32 frames, are provided in the Appendix A for completeness. Regarding the computational efficiency, as it will be visible in the next results, the detection times decrease linearly with the window size since this allows the network to process the input more efficiently, as the tasks are executed in a single forward pass through the network. In addition to these experiments, we also examined the impact of embedding task similarity into the loss function. However, the results showed only a slight 1% deviation from those previously obtained, which does not support any claim of improvement. Consequently, the use of similarity scores, i.e., cosine similarity and Jaccard index, justify the use of shared layers which is already an improvement while obtaining further performance gains may require methodological extensions or additional datasets.

#### 6.2.1. Car Hacking Dataset

**Figure A1:**
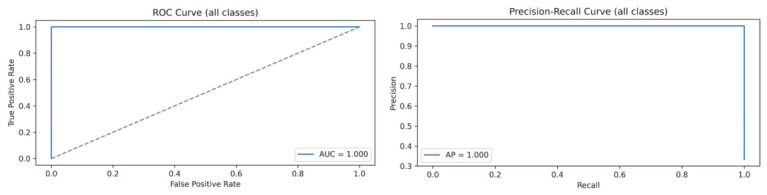
ROC and PR curves for attacks detected in the MTL stage—1 CAN frame/window—Car Hacking Dataset.

Table 3 summarizes the detection performance and detection time obtained on the Car Hacking dataset for window sizes of 1, 2, and 8 frames. For brevity, the results for 16 and 32 frames per window are deferred to Table A1 from Appendix A. The ROC and PR curves obtained for a window size of 1 frame are also illustrated in Figure A1 from the Appendix A.

Flooding attacks are detected in the first stage, analogous to signature-based detection (frames with ID = 0x000), achieving a 100% detection rate. Frames with IDs that differ from the legitimate ones are also flagged as fuzzing attacks in the first stage. The remaining fuzzing attacks with legitimate IDs are then fed to our model. This explains why Table 3 lists three rows for fuzzing: one for the filtering stage, one for the MTL, and one presenting the aggregated results from both stages, which, in fact, represents the overall performance of the IDS. For a single-frame window, the model achieves perfect detection performance, with an overall recall close to 100%, a false positive rate of 0.00%, and R-AUC and PR–AUC values nearing 100% for both fuzzing and spoofing attacks. However, this configuration requires the longest detection time, reaching 334.24 s for the entire testing trace of 4,735,744 frames that entered into the MTL model (96,498 frames were already filtered in the first stage).

When using a window size of 2 frames, the detection rate is almost 100% in terms of recall, while the detection time is 244.57 s for the same testing set. This demonstrates that doubling the window size improves the computational efficiency, without compromising the detection accuracy. For a window size set to 8 frames, the model maintains high detection performance with very low false positive rates, while reducing the detection time to 42.11 s, corresponding to a speedup of nearly 7.93 times compared to single-frame detection. These results confirm that an 8-frame window provides the optimal balance between detection accuracy and efficiency for the Car Hacking dataset.

#### 6.2.2. Survival Analysis Dataset

**Figure A2:**
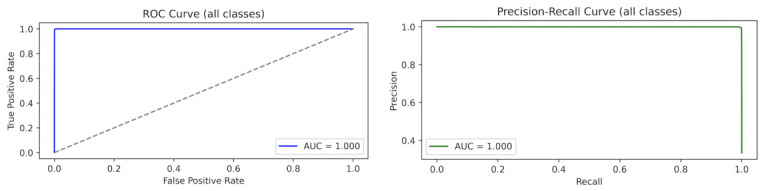
ROC and PR curves for attacks detected in the MTL stage—1 CAN frame/window—Survival Analysis Dataset.

Table 4 reports the detection performance and inference times obtained on the Survival Analysis dataset for window sizes of 1, 2, and 8 frames after 50 training epochs. For brevity, the results for 16 and 32 frames per window are deferred to Table A2 from Appendix A. Figure A2 from the Appendix A presents the ROC and PR curve results for window size of one frame.

All flooding and fuzzing attacks are filtered in the first stage, following the approach used in the Car Hacking dataset. The IDS achieves strong overall detection performance for a single-frame window, with a total recall of 97.59% and a false positive rate of 0.01%. Malfunction attacks are detected with perfect recall, while for fuzzing attacks, the aggregated recall is around 85%. Indeed, the MTL model achieves only a recall of 22%, which is understandable since most of the fuzzing frames were already detected in the first stage and only a few frames passed through the filter and reached the MTL stage. As shown in row 4 of Table 4, the testing set includes only 100 fuzzing attacks classified by the MTL. Consequently, the model cannot learn effectively, as too few fuzzing attacks are also available in the training set. This configuration requires the longest detection time, reaching 0.96 s for the entire testing trace of 36,767 frames that entered into the MTL model (1062 frames were already filtered in the first stage). When increasing the window size to 2 frames, the overall recall remains high at 97.31%, with a false positive rate of 0.06%. Again, the MTL model exhibits a recall of only 1.04% for fuzzing attacks due to the reduced number of attack samples, only 100 frames, reaching the MTL stage. At the same time, the inference time is reduced to 0.49 s, achieving a speedup of approximately 1.71 times compared with the STL baseline and approximately 1.95 times compared with the single-frame window. For a window size of 8 frames, the inference time further decreases to 0.14 s, corresponding to a speedup of approximately 1.78 times compared with the STL baseline and approximately 6.85 times compared with the single-frame window. The overall recall remains high at 96.73%, while fuzzing attacks continue to be detected reliably in the aggregated evaluation, despite the poor recall of the MTL stage that is due to the low number of samples that reached it.

#### 6.2.3. Cloud IDS Dataset

**Figure A3:**
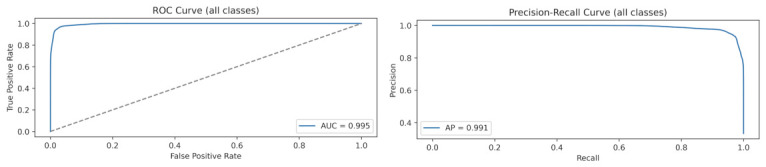
ROC and PR curves for attacks detected in the MTL stage—1 CAN frame/window—Cloud IDS Dataset.

Table 5 reports the detection performance and inference time obtained on the Cloud IDS dataset for window sizes of 1, 2, and 8 frames. For brevity, results for larger window sizes (16 and 32 frames) are deferred in Table A3 from the Appendix A. Also, the ROC and PR curves obtained for a window of 1 frame are shown in Figure A3 in the Appendix A.

For a single-frame window, the model achieves moderate detection performance, with a total recall of 69.27% and a lower false positive rate of 0.09%. The inference time for the full test sequence processed by the MTL model is 4.20 s. Fuzzing attacks are detected nearly perfectly, with a recall of 99.97% and a precision of 100%, while replay attacks show a lower recall of 56.55% due to their similarity with legitimate frames. When using a window size of 2 frames, the total recall improves to 80.94%. Recall for replay attacks increases to 72.79%, and precision decreases slightly due to fewer false positives. The inference time decreases to 2.34 s, benefiting from reduced forward passes. Using an 8-frame window, the overall detection improves significantly, with a recall of up to 95.56%. This happens because replay attacks are detected with 93.95% recall, while fuzzing detection remains nearly perfect. The inference time drops sharply to 0.64 s, corresponding to a speedup of approximately 6.56 times compared to the single-frame configuration and of approximately 1.82 compared to STL scenario.

#### 6.2.4. Our Dataset

Table 6 summarizes the detection performance and inference time obtained on our dataset for window sizes of 1, 2, and 8 frames. The evaluation reports both per-attack and overall performance metrics. Extended results for larger window sizes (16 and 32 frames) are presented in Table A4 from the Appendix A.

**Figure 9:**
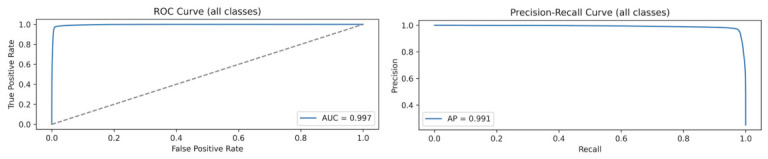
ROC and PR curves for attacks detected in the MTL stage—1 CAN frame/window—Our Dataset.

For a single-frame window, the model achieves strong detection performance across most attack types, with an overall recall of 88.15% and a false positive rate of 0.07%. Fuzzing and flooding attacks are completely detected at the filtering stage, since the fuzzing attacks in our dataset are launched with IDs outside the legitimate traffic IDs. Malfunction and spoofing attacks are detected with near-perfect recall by the MTL, while replay attacks exhibit lower recall due to their similarity to genuine traffic patterns. The MTL inference time for this configuration is 7.71 s, corresponding to a speedup of approximately 1.44 times compared to the STL baseline, which requires 11.16 s. The ROC and PR curves, depicted in Figure 9, indicate high performance, with ROC–AUC = 99.70% and PR–AUC = 99.10%, demonstrating strong class separability and robust detection capability even without extended temporal context. When using a window size of 2 frames, the overall recall improves to 91.52%, while maintaining a low false positive rate of 0.09%. Detection performance increases particularly for replay attacks, whose recall rises noticeably compared to the single-frame case. In addition, the inference time is reduced to 3.48 s, achieving a speedup of approximately 2.07 times over the STL model. This highlights the benefit of moderate temporal aggregation in improving both accuracy and computational efficiency. For a window size of 8 frames, the model further reduces the inference time to 0.84 s, corresponding to a speedup of approximately 1.86 times compared to the STL baseline. The overall recall remains high at 89.24%, with a slight increase in the false positive rate to 0.32%, mainly attributed to replay and spoofing attacks. These results indicate again that an 8-frame window offers a favorable trade-off between inference speed and detection performance for our dataset.

**Figure 10:**
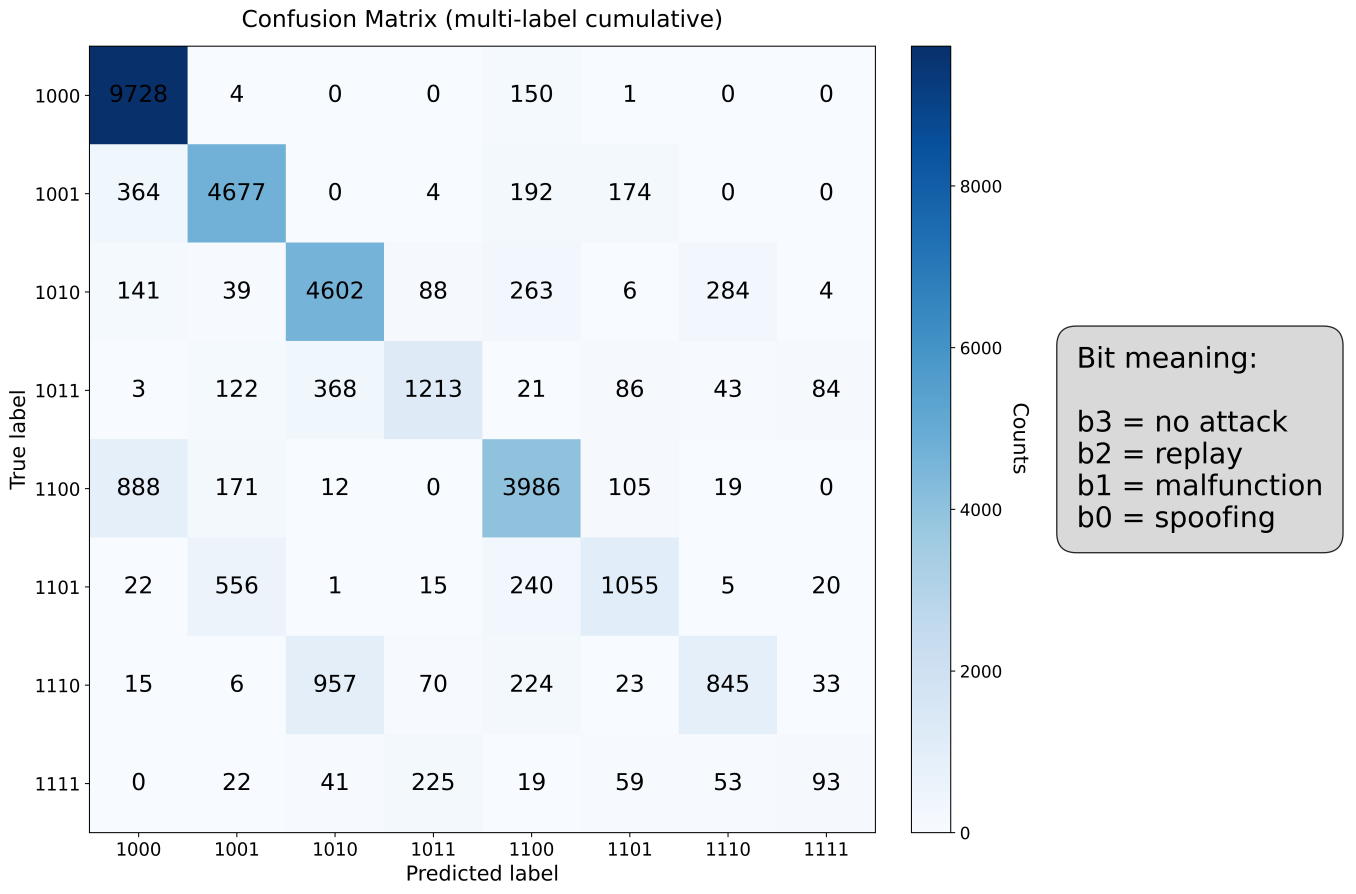
Confusion Matrix—Multi-label Cumulative—8 CAN frames/window—Our Dataset.

For the evaluation of multi-label classification over 8-frame windows, in Figure 10 each sample is represented by a 4-bit binary vector with bits ordered as: 1st bit for no_attack, 2nd for replay, 3rd for malfunction, and 4th for spoofed, where a value of 1 indicates that the corresponding class occurs at least once within the considered window, while a value of 0 indicates its absence. For example, 1000 indicates genuine activity only, while 1010 indicates the coexistence of genuine activity and malfunction. Similar interpretations apply to the remaining class combinations.

The confusion matrix shows a predominantly diagonal distribution, indicating that the model correctly recognizes most multi-label patterns in the dataset. High true-positive rates for classes such as 1000, 1001, 1010, and 1100 demonstrate strong discrimination of common temporal configurations. Most misclassifications occur between classes with similar binary structures, particularly those sharing the no_attack bit, suggesting partial overlap in their feature representations over time. Despite these localized confusions, the overall matrix reflects strong generalization in the multi-window setting, confirming that the model effectively captures and distinguishes combined attack states.

In the following, we present comparisons of our two-stage IDS against existing methods across different datasets. We choose to compare the recall metric, as it provides an overview of the IDS capability to detect adversarial manipulations. The results highlight detection performance and efficiency for various attack types and window sizes. On the Car Hacking dataset, as presented in Table 7, our method achieves 100% recall for flooding and spoofing attacks across all window sizes (w = 1, w = 2, w = 8), outperforming or matching the best results from previous works: flooding (96.10% in [29], 100% in [55,56]) and spoofing (96.00–96.20% in [29], 93.07–93.52% in [55], 96.00–100.00% in [56]). For the fuzzing attack, our recall values are 98.13% (w = 1), 98.11% (w = 2), and 97.95% (w = 8), which are very close to the top reported 99.00% in [56].

On the Survival Analysis dataset, as presented in Table 8, our method achieves 100% recall for malfunction (w = 1 and w = 2) and flooding (w = 1, w = 2 and w = 8). For fuzzing, the recall is 85.90% (w = 1), 84.50% (w = 2), and 85.30% (w = 8). With the exception of fuzzing, these results are comparable with the ones from [57] (malfunction 99.50–99.78%, fuzzing 98.36–99.39%, flooding 99.91–100%) and [58] (malfunction 99.80%, fuzzing 99.54%, flooding 99.95%), while clearly outperforming [59] (malfunction 61.45–73.69%, fuzzing 71.42–81.89%, flooding 65.9–76.99%). For fuzzing the lesson learned is that while the filtering approach was highly effective on the other two datasets, here it actually lowers the detection ability of the deep-learning layer as fuzzing attacks are largely absent from the training samples.

On the Cloud IDS dataset, as presented in Table 9, our method achieves very high recall for fuzzing (99.97% for w = 1 and w = 2, 98.81% for w = 8) and for replay attacks, recall increases with the window size (56.55% for w = 1, 72.79% for w = 2, 93.95% for w = 8), showing clear benefits of larger windows for complex attacks. Compared to [2] (fuzzing 99.00%, replay 59.00–96.00%), our method provides superior detection for fuzzing and comparable detection for replay.

Although detection time and model complexity are relevant comparison metrics, direct comparisons with related works are limited because runtime results are often obtained under different hardware, software, and experimental setups. In addition, detailed information on model complexity, such as the number of parameters or computational cost, is not reported consistently across related studies. Therefore, computational efficiency in this work is evaluated through controlled comparisons between the proposed multitask learning (MTL) model and its STL counterpart under identical experimental conditions, providing a reliable assessment of the efficiency gains introduced by the proposed architecture.

## 7. Conclusions

The proposed methodology benefits from a clear computational gain, which is due to the use of a larger window size. By corroborating the detection metrics with the computational gains, a windows of 8 frames seems to be optimal. This means that in a cloud-based scenario a window of 512 bits, that is 8 CAN frames, can be send periodically from the vehicle to the cloud for on-line evaluation. The detection rates from our work are in-line with the results that are already reported in other works, in this respect, the advantages of multitask learning are especially in the computational time and the flexibility of detecting and classifying several attacks in the same window. The use of more complex neural network architectures combined with dedicated GPUs is a promising approach for future work. In addition, federated learning may be leveraged to mitigate data privacy concerns. Model compression techniques should also be explored to facilitate deployment on resource-constrained local ECUs. Furthermore, investigating advanced architectures such as CNNs, LSTMs, or Transformers may lead to improved detection performance. The public availability of the dataset enables future research to explore additional detection methods on the collected data.

## Figures and Tables

**Table 1:** Attack datasets used in our evaluation.

Attack Types	Car Hacking Dataset [48]	Survival Analysis Dataset [49]	Cloud IDS Dataset [2]	Our Dataset
malfunction	-	injections with a targeted ID and 0x00 data bytes or randomly generated datafield	-	injections with a targeted ID and randomly generated datafield using a fixed attack probability
fuzzing	injections with randomly generated ID and datafield every 0.5 ms	injections with randomly generated ID and datafield every 0.3 ms	injections with a targeted ID and randomly generated datafield using a fixed attack probability	injections with randomly generated ID (outside the set of legitimate IDs) and datafield using a fixed attack probability
spoofing (gear, RPM, speed)	injections with a targeted ID carrying specific signals (gear, RPM) and a fixed value assigned to them every 1 ms	-	-	injections with a targeted ID carrying a specific signal (vehicle speed) and a randomly altered value assigned to it, using a fixed attack probability
replay	-	-	injections with a targeted ID and same datafield with the previously received frame and using a fixed attack probability	injections with a targeted ID and same datafield with the previously received frame using a fixed attack probability
flooding (DoS)	injections with ID 0x000 every 0.3 ms	injections with ID 0x000 at random points of time	-	bursts of 10 injections with ID 0x000, each separated by 150 μs

**Table 2:** Training Hyperparameters.

Component	Value
Optimizer	AdamW
Learning rate	0.001
Weight decay	1 × 10^−5^
Batch size	32
Epochs	50
Loss function	Binary cross-entropy
Activation functions	Swish/LeakyReLU
Dropout	0.2–0.5
Validation split	10%
Sequence length	1, 2, 8, 16 and 32
Feature scaling	StandardScaler
Callback	ReduceLROnPlateau

**Table 3:** Detection results and detection times—50 training epochs—Car Hacking Dataset.

		Evaluation Metrics for Detection Performance							Detection Time	
Window	Att. Type	TP	TN	FP	FN	TNR	FPR	Recall	MTL	STL
w = 1	Fuzzing (agg.)	98,366	9,467,680	64	1876	100.00	0.00	98.13	334.24 s (1.71× faster)	574.26 s
	Fuzzing (filter)	96,498	4,733,872	0	1872	100.00	0.00	98.10		
	Fuzzing (MTL)	1868	4,733,808	64	4	100.00	0.00	99.79		
	Spoofing	250,431	4,485,313	0	0	100.00	0.00	100.00		
	Flooding (filter)	117,504	615,650	0	0	100.00	0.00	100.00		
	**Total**	466,301	14,568,643	64	1876	100.00	0.00	99.60		
w = 2	Fuzzing (agg.)	98,334	7,099,824	65	1891	100.00	0.00	98.11	244.57 s (1.15× faster)	282.84 s
	Fuzzing (filter)	96,498	4,733,872	0	1872	100.00	0.00	98.10		
	Fuzzing (MTL)	1836	2,365,952	65	19	100.00	0.00	98.98		
	Spoofing	213,770	2,154,102	0	0	100.00	0.00	100.00		
	Flooding (filter)	117,504	615,650	0	0	100.00	0.00	100.00		
	**Total**	429,608	9,869,576	65	1891	100.00	0.00	99.56		
w = 8	Fuzzing (agg.)	98,110	5,323,961	88	2051	100.00	0.00	97.95	42.11 s (2.42× faster)	102.07 s
	Fuzzing (filter)	96,498	4,733,872	0	1872	100.00	0.00	98.10		
	Fuzzing (MTL)	1612	590,089	88	179	99.99	0.01	90.01		
	Spoofing	96,021	495,923	22	2	100.00	0.00	100.00		
	Flooding (filter)	117,504	615,650	0	0	100.00	0.00	100.00		
	**Total**	311,635	6,435,534	110	2053	100.00	0.00	99.35		

**Table 4:** Detection results and detection times—50 training epochs—Survival Analysis Dataset.

		Evaluation Metrics for Detection Performance							Detection Time	
Window	Att. Type	TP	TN	FP	FN	TNR	FPR	Recall	MTL	STL
w = 1	Malfunction	1610	35,155	2	0	99.99	0.01	100.00	0.96 s (1.73× faster)	1.67 s
	Fuzzing (agg.)	1084	73,320	14	178	99.98	0.02	85.90		
	Fuzzing (filter)	1062	36,667	0	100	100.00	0.00	91.39		
	Fuzzing (MTL)	22	36,653	14	78	99.96	0.04	22.00		
	Flooding (filter)	4517	19,596	0	0	100.00	0.00	100.00		
	**Total**	7211	128,071	16	178	99.99	0.01	97.59		
w = 2	Malfunction	1474	16,857	52	0	99.69	0.31	100.00	0.49 s (1.71× faster)	0.84 s
	Fuzzing (agg.)	1063	54,952	2	195	100.00	0.00	84.50		
	Fuzzing (filter)	1062	36,667	0	100	100.00	0.00	91.39		
	Fuzzing (MTL)	1	18,285	2	95	99.99	0.01	1.04		
	Flooding (filter)	4517	19,596	0	0	100.00	0.00	100.00		
	**Total**	7054	91,405	54	195	99.94	0.06	97.31		
w = 8	Malfunction	745	3662	157	31	95.89	4.11	96.01	0.14 s (1.78× faster)	0.25 s
	Fuzzing (agg.)	1062	41,179	0	183	100.00	0.00	85.30		
	Fuzzing (filter)	1062	36,667	0	100	100.00	0.00	91.39		
	Fuzzing (MTL)	0	4512	0	83	100.00	0.00	0.00		
	Flooding (filter)	4517	19,596	0	0	100.00	0.00	100.00		
	**Total**	6324	64,437	157	214	99.76	0.24	96.73		

**Table 5:** Detection results and detection times—50 training epochs—Cloud IDS Dataset.

		Evaluation Metrics for Detection Performance							Detection Time	
Window	Att. Type	TP	TN	FP	FN	TNR	FPR	Recall	MTL	STL
w = 1	Fuzzing	11,421	253,198	0	3	100.00	0.00	99.97	4.20 s (1.84× faster)	7.76 s
	Replay	15,599	236,582	456	11,985	99.81	0.19	56.55		
	**Total**	27,020	489,780	456	11,988	99.91	0.09	69.27		
w = 2	Fuzzing	11,239	121,069	0	3	100.00	0.00	99.97	2.34 s (1.82× faster)	4.27 s
	Replay	19,100	105,217	854	7140	99.19	0.81	72.79		
	**Total**	30,339	226,286	854	7143	99.62	0.38	80.94		
w = 8	Fuzzing	10,171	22,777	6	123	99.97	0.03	98.81	0.64 s (1.82× faster)	1.17 s
	Replay	19,451	11,705	668	1253	94.60	5.40	93.95		
	**Total**	29,622	34,482	674	1376	98.08	1.92	95.56		

**Table 6:** Detection results and detection times—50 training epochs—Our Dataset.

		Evaluation Metrics for Detection Performance							Detection Time	
Window	Att. Type	TP	TN	FP	FN	TNR	FPR	Recall	MTL	STL
w = 1	Malfunction	11,486	247,962	1	80	100.00	0.00	99.31	7.71 s (1.44× faster)	11.16 s
	Fuzzing (filter)	11,289	269,018	0	0	100.00	0.00	100.00		
	Spoofing	10,868	248,413	3	245	100.00	0.00	97.80		
	Replay	5130	247,359	876	6164	99.65	0.35	45.42		
	Flooding (filter)	9490	270,817	0	0	100.00	0.00	100.00		
	**Total**	48,263	1,283,569	880	6489	99.93	0.07	88.15		
w = 2	Malfunction	11,134	118,488	7	135	99.99	0.01	98.80	3.48 s (2.07× faster)	7.22 s
	Fuzzing (filter)	11,289	269,018	0	0	100.00	0.00	100.00		
	Spoofing	10,578	118,793	11	382	99.99	0.01	96.51		
	Replay	6931	117,980	788	4065	99.34	0.66	63.03		
	Flooding (filter)	9490	270,817	0	0	100.00	0.00	100.00		
	**Total**	49,422	895,096	806	4582	99.91	0.09	91.52		
w = 8	Malfunction	9003	22,313	76	1049	99.66	0.34	89.56	0.84 s (1.86× faster)	1.57 s
	Fuzzing (filter)	11,289	269,018	0	0	100.00	0.00	100.00		
	Spoofing	8405	22,114	550	1372	97.57	2.43	85.97		
	Replay	6779	21,353	1308	3001	94.23	5.77	69.31		
	Flooding (filter)	9490	270,817	0	0	100.00	0.00	100.00		
	**Total**	44,966	605,615	1934	5422	99.68	0.32	89.24		

**Table 7:** Detection performance in terms of recall—our solution versus related works on the Car Hacking dataset.

Wrk.	Fuzzing	Spoofing (Gear & RPM)	Flooding
[29]	95.40	96.00–96.20	96.10
[55]	97.55	93.07–93.52	100.00
[56]	99.00	96.00–100.00	100.00
our (w = 1)	98.13	100.00	100.00
our (w = 2)	98.11	100.00	100.00
our (w = 8)	97.95	100.00	100.00

**Table 8:** Detection performance in terms of recall—our solution versus related works on the Survival Analysis dataset.

Wrk.	Malfunction	Fuzzing	Flooding
[57]	99.50–99.78	98.36–99.39	99.91–100.00
[58]	99.80	99.54	99.95
[59]	61.45–73.69	71.42–81.89	65.90–76.99
our (w = 1)	100.00	85.90	100.00
our (w = 2)	100.00	84.50	100.00
our (w = 8)	96.01	85.30	100.00

**Table 9:** Detection performance in terms of recall—our solution versus related works on the Cloud IDS Dataset.

Wrk.	Fuzzing	Replay
[2]	99.00	59.00–96.00
our (w = 1)	99.97	56.55
our (w = 2)	99.97	72.79
our (w = 8)	98.81	93.95

## Data Availability

The raw data supporting the conclusions of this article will be made available by the authors on request. Data is also available at the link (https://www.aut.upt.ro/~bgroza/projects/mtl-ids/combined_output_dataset.zip).

## Appendix A

For the Car Hacking dataset, as can be shown in Table A1, increasing the window size to 16 frames results in a total recall of 99.04% with a false positive rate of 0.00%, while significantly reducing the MTL inference time to 22.67 s, corresponding to a 2.00 speedup compared to STL scenario. Further increasing the window size to 32 frames leads to a slight decrease in total recall to 98.87% and a marginal increase in FPR to 0.01%, while the inference time is reduced to 12.92 s, achieving a 1.87 speedup.

**Table A1:** Detection results and detection times—50 training epochs—Car Hacking Dataset.

		Evaluation Metrics for Detection Performance							Detection Time	
Window	Att. Type	TP	TN	FP	FN	TNR	FPR	Recall	MTL	STL
w = 16	Fuzzing (agg.)	97,687	5,027,983	164	2392	100.00	0.00	97.61	22.67 s (2.00× faster)	45.37 s
	Fuzzing (filter)	96,498	4,733,872	0	1872	100.00	0.00	98.10		
	Fuzzing (MTL)	1189	294,111	164	520	99.94	0.06	69.57		
	Spoofing	49,369	246,362	91	162	99.96	0.04	99.67		
	Flooding (filter)	117,504	615,650	0	0	100.00	0.00	100.00		
	**Total**	264,560	5,889,995	255	2554	100.00	0.00	99.04		
w = 32	Fuzzing (agg.)	97,270	4,879,943	364	2657	99.99	0.01	97.34	12.92 s (1.87× faster)	24.24 s
	Fuzzing (filter)	96,498	4,733,872	0	1872	100.00	0.00	98.10		
	Fuzzing (MTL)	772	146,071	364	785	99.75	0.25	49.58		
	Spoofing	24,756	123,146	20	70	99.98	0.02	99.72		
	Flooding (filter)	117,504	615,650	0	0	100.00	0.00	100.00		
	**Total**	239,530	5,618,739	384	2727	99.99	0.01	98.87		

For a single-frame window, both ROC and PR curves indicate perfect separability, with ROC–AUC and PR–AUC equal to 100%, confirming the strong discriminative capability of the model for this dataset, as depicted in Figure A1.

Table A2 presents the results for the Survival Analysis dataset, where a 16-frame window achieves a total recall of 96.96% with an FPR of 0.02%, and an inference time of 0.08 s, corresponding to a 2.12 speedup over the STL baseline. Using a 32-frame window slightly improves the total recall to 97.29%, while further reducing the inference time to 0.05 s, resulting in a 2.40 speedup compared to STL scenario.

**Table A2:** Detection results and detection times—50 training epochs—Survival Analysis Dataset.

		Evaluation Metrics for Detection Performance							Detection Time	
Window	Att. Type	TP	TN	FP	FN	TNR	FPR	Recall	MTL	STL
w = 16	Malfunction	383	1894	11	9	99.42	0.58	97.70	0.08 s (2.12× faster)	0.17 s
	Fuzzing (agg.)	1062	20,756	0	178	100.00	0.00	85.65		
	Fuzzing (filter)	1062	36,667	0	100	100.00	0.00	91.39		
	Fuzzing (MTL)	0	1088	0	60	100.00	0.00	0.00		
	Flooding (filter)	4517	19,596	0	0	100.00	0.00	100.00		
	**Total**	5962	60,376	11	187	99.98	0.02	96.96		
w = 32	Malfunction	195	952	0	1	100.00	0.00	99.49	0.05 s (2.40× faster)	0.12 s
	Fuzzing (agg.)	1062	37,755	0	160	100.00	0.00	86.91		
	Fuzzing (filter)	1062	36,667	0	100	100.00	0.00	91.39		
	Fuzzing (MTL)	0	1088	0	60	100.00	0.00	0.00		
	Flooding (filter)	4517	19,596	0	0	100.00	0.00	100.00		
	**Total**	5774	58,303	0	161	100.00	0.00	97.29		

Similar to the Car Hacking dataset, the single-frame configuration yields perfect ROC and PR curves, with ROC–AUC and PR–AUC values of 100%, as illustrated in Figure A2.

For the Cloud IDS dataset the detection results are reported in Table A3. The use of a 16-frame window yields a total recall of 97.57% with an FPR of 3.60%, and reduces the MTL inference time to 0.48 s, corresponding to a 1.52 speedup relative to the STL baseline. Increasing the window size to 32 frames further reduces the inference time to 0.29 s, achieving a 1.44 speedup, although the false positive rate increases to 18.72%, particularly due to the more challenging detection of replay attacks.

For the single-frame setting, the ROC–AUC and PR–AUC reach 99.50% and 99.10%, respectively, indicating strong performance despite the higher class imbalance and attack similarity, as shown in Figure A3.

**Table A3:** Detection results and detection times—50 training epochs—Cloud IDS Dataset.

		Evaluation Metrics for Detection Performance							Detection Time	
Window	Att. Type	TP	TN	FP	FN	TNR	FPR	Recall	MTL	STL
w = 16	Fuzzing	8692	7588	26	232	99.66	0.34	97.40	0.48 s (1.52× faster)	0.73 s
	Replay	14,039	1840	326	333	84.95	15.05	97.68		
	**Total**	22,731	9428	352	565	96.40	3.60	97.57		
w = 32	Fuzzing	6360	1431	241	237	85.59	14.41	96.41	0.29 s (1.44× faster)	0.42 s
	Replay	8133	19	93	24	16.96	83.04	99.71		
	**Total**	14,493	1450	334	261	81.28	18.72	98.23		

Last but not least, the results for our dataset are reported in Table A4. As can be seen, when using a 16-frame window, the model achieves a total recall of 91.03% with an FPR of 0.73%, while reducing the MTL inference time to 0.51 s, corresponding to a 1.82 speedup compared to STL baseline. Using a 32-frame window further decreases the inference time to 0.28 s, achieving a 1.92 speedup, while maintaining a comparable total recall of 95.86%. These results indicate that larger temporal windows primarily improve computational efficiency, while detection performance remains stable.

**Table A4:** Detection results and detection times—50 training epochs—Our Dataset.

		Evaluation Metrics for Detection Performance							Detection Time	
Window	Att. Type	TP	TN	FP	FN	TNR	FPR	Recall	MTL	STL
w = 16	Malfunction	7129	7035	641	1415	91.65	8.35	83.44	0.51 s (1.82× faster)	0.93 s
	Fuzzing (filter)	11,289	269,018	0	0	100.00	0.00	100.00		
	Spoofing	7257	6755	1137	1071	85.59	14.41	87.14		
	Replay	6717	5552	2312	1639	70.60	29.40	80.39		
	Flooding (filter)	9490	270,817	0	0	100.00	0.00	100.00		
	**Total**	41,882	559,177	4090	4125	99.27	0.73	91.03		
w = 32	Malfunction	5586	971	865	688	52.89	47.11	89.03	0.28 s (1.92× faster)	0.54 s
	Fuzzing (filter)	11,289	269,018	0	0	100.00	0.00	100.00		
	Spoofing	5691	810	1116	493	42.06	57.94	92.03		
	Replay	5755	437	1465	453	22.98	77.02	92.70		
	Flooding (filter)	9490	270,817	0	0	100.00	0.00	100.00		
	**Total**	37,811	542,053	3446	1634	99.37	0.63	95.86		
